# Utility of untimed single urine protein/creatinine ratio as a substitute for 24-h proteinuria for assessment of proteinuria in systemic lupus erythematosus

**DOI:** 10.1186/s13075-015-0808-x

**Published:** 2015-10-24

**Authors:** Jorge Medina-Rosas, Dafna D. Gladman, Jiandong Su, Arthy Sabapathy, Murray B. Urowitz, Zahi Touma

**Affiliations:** Clinical and Research Fellow, University of Toronto Lupus Clinic, Toronto Western Hospital, Centre for Prognosis Studies in the Rheumatic Diseases, Toronto, ON Canada; Toronto Western Research Institute, University of Toronto, Toronto, ON Canada; University of Toronto Lupus Clinic, Centre for Prognosis Studies in the Rheumatic Diseases, Toronto Western Hospital, University of Toronto, Toronto, ON Canada; University of Toronto Lupus Clinic, Toronto Western Hospital, Centre for Prognosis Studies on the Rheumatic Diseases, Toronto, ON Canada

**Keywords:** Systematic lupus erythematosus, Lupus nephritis, Kidney

## Abstract

**Introduction:**

In this study, we determined: (1) the utility of an untimed sample of urine protein/creatinine ratio (PCR) as a screening test for proteinuria, (2) its ability to accurately measure proteinuria, and (3) cutoff values for PCR predicting protein content in a 24-h urine collection sample (24hP) of 0.5, 1.0, and 2.0 g/day.

**Methods:**

Analysis was performed on data from a single lupus cohort (2008–2014). Proteinuria was measured in a 24hP and with PCR. On the basis of 24hP, samples were divided into 4 groups: group 1, <0.5 g/day; group 2, 0.5–0.99 g/day; group 3, 1–1.99 g/day; and group 4, ≥2 g/day. To determine the validity of PCR in screening for proteinuria, the Pearson correlation coefficient was determined for the urine samples with normal PCR (<0.05 g/mmol) and normal 24hP (<0.5 g/day). The sensitivity, specificity, positive predictive value (PPV), and negative predictive value (NPV) of PCR were calculated. To determine the ability of PCR to accurately measure the level of proteinuria, in addition to the correlation between 24hP and PCR, agreement was determined by intraclass correlation coefficient, concordance correlation coefficient, and Bland-Altman plot between 24hP/24hC and PCR. The best cutoffs for PCR predicting a 24hP of 0.5, 1.0, and 2.0 g/day were determined with the receiver operating characteristic curve.

**Results:**

The correlation of the samples with normal PCR as well as 24hP (n = 552) was 0.29 (p < 0.0001). PCR sensitivity and specificity against 24hP were 91 % and 83 %, respectively. The PPV was 82.5 %, and the NPV was 91.4 %. The correlation for all samples (n = 1233) was high, but low to moderate for groups 1, 2, 3, and 4. The agreement for all samples was appropriate but poor for groups 1, 2, 3, and 4. PCR cutoffs for 24hP of 0.5, 1.0, and 2.0 g/day were 0.08, 0.16, and 0.35 g/mmol, respectively.

**Conclusions:**

PCR can be used as a screening test for proteinuria, and the best cutoff value to predict a 24hP of 0.5 g/day is 0.08 g/mmol (800 mg/g). The accurate level of proteinuria should be measured by the gold standard test, 24hP.

## Introduction

Lupus nephritis (LN) is an immune complex glomerulonephritis with a cumulative incidence of 54 % [1]. LN can occur early (50 %) as well as years after the diagnosis of systemic lupus erythematosus (SLE) is made (45 %) [2]. The manifestations of renal involvement can be diverse, including proteinuria and active urinary sediment [3, 4]. Proteinuria is the most common manifestation of LN, being reported in almost 100 % of patients with LN, followed by granular casts, cellular casts, hematuria, and reduced renal function [1, 5]. Proteinuria is the principal urinary biomarker for the screening of LN [5, 6] and for monitoring disease progression [7–9]. Among the methods for quantification of the proteinuria, the protein content in a 24-h urine collection sample (24hP) has been considered the “gold standard,” but this test is cumbersome for patients and sometimes is collected incorrectly [10]. Ginsberg et al. proposed using the untimed sample of urine protein/creatinine ratio (PCR) instead of the 24hP [11]. Several authors have shown a good correlation between PCR and 24hP in diabetes [12], LN [13, 14], and chronic kidney disease [15]. The American College of Rheumatology (ACR) recommends the PCR for use in clinical trials of LN [16], and the European League Against Rheumatism recommendations for the management of adult and pediatric LN suggest using the first morning PCR as a valid measure of proteinuria [17]. Nevertheless, other studies have shown a weak agreement between PCR and 24hP results [18–20]. Clearly, there is no agreement between the existing studies on the utility of PCR. The variance of the results of the published studies is partly related to the method of collection of PCR and the use of inappropriate statistical analyses in various studies. The aim of this study was to determine (1) the utility of the spot urine protein/creatinine ratio as a screening test for proteinuria in patients with SLE, (2) the ability of PCR to accurately measure the level of proteinuria, and (3) the cutoff values for PCR predicting 24hP of 0.5, 1.0, and 2.0 g/day.

## Methods

### Subjects and assessment

#### Patient selection

All adult (≥18 years old) patients with lupus at the University of Toronto Lupus Clinic at the Toronto Western Hospital who fulfilled at least four of the ACR revised criteria for the classification of SLE [21] or who met three criteria and had a typical biopsy lesion of SLE were studied.

#### Inclusion criteria

All patients’ results available in the data collected from May 2008 until December 2014 were screened for inclusion in this study. Patient visits with proteinuria assessment documented by PCR and 24hP, with both tests being done within 4 days, were identified and analyzed.

#### Exclusion criteria

We excluded (1) patients with estimated glomerular filtration rate (GFR) or creatinine clearance (calculated from the 24-h urine collection) <15 ml/min, (2) patients on dialysis, (3) patients with proteinuria not related to LN (e.g., diabetes mellitus, chronic sclerosing LN, and others), and (4) patients with kidney transplantation. The majority (>90 %) of urine specimens were handled and interpreted at one laboratory, and the appropriate measurements of preservation and shipment of the urine samples were applied.

#### Assessment of patients and measurement of proteinuria

We conducted a retrospective analysis of data collected prospectively from May 2008 to December 2014. Patients were attending the lupus clinic at 2- to 6-month intervals regardless of the activity of their disease. Patients’ assessments include complete history, physical examination, information about drug treatment, and laboratory examinations. For this study, the laboratory results including GFR and serum creatinine were analyzed. The PCR samples were taken the day of the follow-up visit in the clinic (between 9:00 am and 5:00 pm), and the results were reviewed. If PCR was abnormal (>0.05 g/mmol), patients are contacted and instructed to collect a 24-h urine sample starting the next day. The 24-h urine sample and PCR were also collected at follow-up visits for all patients with proteinuria to check on the response to therapy. The 24-h urine samples were collected as follows: Patients were instructed to empty the bladder in the morning and discard the urine, and from that point onward for 24 h, all urine was to be saved in the container. At the end of that 24-h period, the bladder was emptied, and that urine was saved.

The collection, storage, and use of the clinical and laboratory data on the patients at the center were conducted in accordance with the Declaration of Helsinki and were approved by the Research Ethics Board of the University Health Network, Toronto, ON, Canada. All patients signed an informed consent form before participation.

#### Adequacy of urine collection

The adequacy of the 24-h urine sample was assessed by comparing the creatinine from the 24-h urine sample collection (24hC) with the expected creatinine content (ExC) using the following the formula: ExC = 28 − (0.2 × age) × weight (kg) in men and 23.8 − (0.17 × age) × weight in women [22]. A stratification for ExC by ethnicity was performed because it is likely that the amount of ExC varies by ethnicity in addition to body weight and/or muscle mass. Undercollection was defined as a ratio of (ExC − 24hC)/ExC >0.2, and those samples were excluded [20, 23]. GFR was calculated using the Chronic Kidney Disease Epidemiology Collaboration equation [24]. Patients were also screened to ensure they were not taking any drugs that would affect the creatinine clearance, such as trimethoprim.

#### Definitions of outcome measures

Proteinuria was defined as ≥0.5 g/24 day, based on the definitions of the Systemic Lupus Erythematosus Disease Activity Index 2000 (SLEDAI-2K) [25] and the ACR criteria [21]. In the database, proteinuria is recorded as related or unrelated to LN activity. If proteinuria is not attributed to SLE activity, it is not scored as present and does not contribute toward the renal component of SLEDAI-2K. The urinalysis interpretation is based on a physician’s judgment and interpretation of available laboratory and pathologic results of other tests (biopsy, urine culture). The laboratories consider an abnormal PCR to be ≥0.05 g/mmol.

### Statistical analysis

The sample size was estimated using normal approximation [26] and exact methods [27] for design accuracy in diagnostic tests. The sample size in this study (n = 1233) is beyond the minimum needed to detect an expected sensitivity (Sn) of 0.90 and using a minimal accepted Sn of 0.85 (n = 400 by exact method). Patients’ baseline characteristics at the first pair of 24hP and PCR measurements [mean ± standard deviation (SD)] and count (%) were used for continuous and categorical variables, respectively.

Samples were divided into four groups according to protein excretion over 24 h: group 1, >0.5 g/day; group 2, 0.5–0.99 g/day; group 3, 1–1.99 g/day; and group 4, ≥2 g/day.

#### Validity of PCR in screening for proteinuria

The Pearson correlation coefficient was determined for the urine samples with normal PCR (<0.05 g/mmol) and normal 24hP (<0.5 g/day). The Sn, specificity (Sp), positive predictive value (PPV), negative predictive value (NPV), and positive likelihood ratio (LR+) of PCR were calculated, and in this analysis 24hP was considered the external construct. The cutoff for PCR was ≥0.05 g/mmol, and the cutoff for 24hP was ≥0.5 g/day.

#### Correlation

The Pearson correlation coefficient was determined for PCR and 24hP for all urine samples overall and for the four groups. Correlations were interpreted as follows [28]: 0.00–0.29 = negligible, 0.3–0.49 = low, 0.5–0.7 = moderate, 0.7–0.9 = high, and 0.9–0.99 = very high.

#### Agreement

The magnitude of the scales 24hP (g/day) and PCR (g/mmol) is different where 24hP is approximately 7.6 times greater than PCR. Thus, before deriving the agreement between 24hP and PCR, it was essential to adjust for this difference in both scales. For this purpose, the ratio of 24hP and 24hC reported in a 24-h urine sample (24hP/24hC content) was calculated. In the following analyses, we compared 24hP/24hC and PCR.

Intraclass correlation coefficient (ICC) (2, *k*) [29], concordance correlation coefficient (CCC) [30], and the Bland-Altman plot [31] for PCR and 24hP/24hC were determined. ICC describes the associations and agreement among units in the same group [32]. For the ICC (2, *k*) the first number, 2, designates the model, and *k* signifies the mean of several measurements as the unit of analysis [29, 33]. The results of the ICC (2, *k*) were derived after applying square root transformation of the data. ICC (2, 1) was determined in the stratification analysis by ethnicity. The second number, 1 [ICC (2, 1)], signifies that ICC is calculated using a single measurement 1. ICC ≥0.85 reflects good agreement [34]. The CCC measures the degree to which paired samples fall in the 45-degree line through the origin, being of interest for equivalence of new laboratory methods with the gold standard [30, 35]. CCC <0.9 was considered poor [35]. The Bland-Altman plot provides a visual approach to the data whereby the mean and the difference of PCR and 24hP/24hC were represented and the limits of agreement (±2 SD) were plotted.

#### Determination of the best cutoff values for PCR predicting 24hP of 0.5, 1, and 2 g/day

Two different approaches were used: (1) the best cutoff determined based on a 2 × 2 contingency table (with 24hP considered the gold standard test) and (2) logistic regression analysis. Continuous values of PCR were fed into the model as independent variables, and binary high and low 24hP cutoffs of 0.5, 1.0, or 2.0 were used as dependent variables. The best cutoffs were determined based on the analysis of the receiver operating characteristic (ROC) curve.

#### Sensitivity analysis

We conducted a separate analysis of patients taking and those not taking angiotensin-converting enzyme (ACE) inhibitors and/or angiotensin receptor blockers (ARBs) *p* values <0.05 were defined as statistically significant. The Excel 2010 (Microsoft, Redmond, WA, USA) and SAS 9.3 (SAS Institute, Cary, NC, USA) software programs were used for statistical data analysis.

## Results

### Patient characteristics

A total of 1730 laboratory urine samples from 421 patients were identified. Of these, 497 samples were excluded because of undercollection in 24-h urine samples, and 1233 urine samples from 322 patients included in the final analysis. The ExC values stratified by ethnicity were as follows: white 17.3 ± 2.8 mmol/day, black 17.5 ± 2.2 mmol/day, Asian 17.6 ± 2.6 mmol/day, and other 17.5 ± 2.6 mmol/day. The patients’ demographics are presented in Table 1. The majority of the patients were women (83.5 %) with lupus disease duration of 11.76 ± 9.71 years and age at first 24hP of 40.26 ± 14.76 years. The number of patients in groups 1, 2, 3, and 4 were 208, 42, 29, and 43, respectively. Eight patients (2.5 %) were treated with cyclosporine, three (1.4 %) in group 1, one (2.4 %) in group 2, one (3.4 %) in group 3, and three (7 %) in the group 4. No patients were treated with tacrolimus.

**Table 1:** Demographic characteristics of the patients included in the study

Characteristics	24hP <0.5 g/day	24hP 0.5–0.99 g/day	24hP 1–1.99 g/day	24hP ≥2.0 g/day	Total
Number of samples (%)	662 (53.7 %)	266 (21.6 %)	171 (13.9 %)	174 (14.1 %)	1233 (100 %)
Number of patients (%)	208 (64.6 %)	42 (13.0 %)	29 (9 %)	43 (13.3 %)	322 (100 %)
Females, n (%)	177 (85.1 %)	32 (76.2 %)	23 (79.3 %)	37 (86.0 %)	269 (83.5 %)
Males, n (%)	31 (14.9 %)	10 (23.8 %)	6 (20.7 %)	6 (14.0 %)	53 (16.5 %)
Whites, n (%)	118 (56.7 %)	23 (54.8 %)	11 (37.9 %)	21 (48.8 %)	173 (53.7 %)
Blacks, n (%)	37 (17.8 %)	7 (16.7 %)	6 (20.7 %)	12 (27.9 %)	62 (19.3 %)
Asians, n (%)	21 (10.1 %)	6 (14.3 %)	5 (17.2 %)	4 (9.3 %)	36 (11.2 %)
Other ethnicities, n (%)	32 (15.4 %)	6 (14.3 %)	7 (24.1 %)	6 (14.0 %)	51 (15.8 %)
Disease duration, yr (SD)	13.02 ± 10.38	8.90 ± 6.23	11.54 ± 9.49	8.61 ± 8.07	11.76 ± 9.71
Age at first 24hP, yr (SD)	42.26 ± 14.88	37.02 ± 16.14	38.33 ± 13.66	35.05 ± 11.58	40.26 ± 14.76
Serum creatinine, μmol/L (SD)	84.39 ± 47.30	87.76 ± 42.56	87.76 ± 42.56	80.49 ± 58.02	84.18 ± 47.03
Patients taking prednisone, n (%)	79 (62.7 %)	22 (59.5 %)	16 (59.3 %)	31 (77.5 %)	148 (64.3 %)
Patients taking immunosuppressives, n (%)	70 (55.6 %)	25 (67.6 %)	14 (51.9 %)	28 (70.0 %)	137 (59.6 %)

*24hP* Protein content in a 24-h urine collection sample

### Validity of PCR in screening for proteinuria

Of the 1233 urine samples, 552 samples had normal PCR and 24hP. The Pearson correlation coefficient was 0.29 (*p* < 0.0001). PCR Sn and Sp against 24hP were 91 % and 83 %, respectively. PPV was 82.5 %, NPV was 91.4, and LR+ was 5.4.

### Correlation between PCR and 24hP

**Fig. 1:**
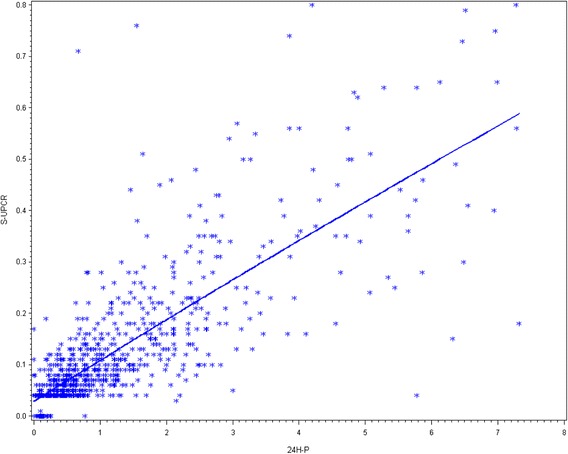
Scatterplot of correlation between protein content in a 24-h urine collection sample; (24hP) and untimed sample of urine protein/creatinine ratio for all urine samples. *PCR* spot urine protein/creatinine ratio

For all samples (n = 1233), the correlation was high (*r* = 0.79) (Fig. 1). However, the correlation was low for group 1 (*r* = 0.4), negligible for group 2 (*r* = 0.2), low for group 3 (*r* = 0.3), and moderate for group 4 was (*r* = 0.6) (all *p* < 0.05) (Table 2). The correlations of both tests stratified by ethnicity were high for all ethnic groups (white 0.76, black 0.86, Asian 0.78, other ethnicities 0.88; all *p* < 0.0001).

**Table 2:** Results of correlations between 24hP and PCR and agreement between 24hP/24hC and PCR

	Correlation	Interpretation^a^	ICC (2, *k*)	Interpretation^b^	CCC	Interpretation^c^
	*p* value		95 % CI		95 % CI	
All samples	0.79	High	0.87	Appropriate	0.85	Poor
	<0.0001		0.83–0.91		0.83–0.87	
24hP	0.4	Low	0.52	Poor	0.62	Poor
<0.5 g/day	<0.0001		0.50–0.53		0.56–0.67	
24hP	0.2	Negligible	0.57	Poor	0.34	Poor
0.5–0.99 g/day	0.0007		0.55–0.58		0.24–0.46	
24hP	0.3	Low	0.73	Poor	0.55	Poor
1–1.99 g/day	0.001		0.70–0.76		0.43–0.68	
24hP	0.6	Moderate	0.77	Poor	0.44	Poor
≥2.0 g/day	0.0001		0.70–0.83		0.35–0.53	

*CCC* concordance correlation coefficient, *CI* confidence interval, 24hC measured creatinine content in a 24-h urine sample collection, *24hP* protein content in a 24-h urine collection sample, *ICC* intraclass correlation coefficient

The ratio of the protein content and the creatinine content in a 24-h urine collection sample (24hP/24hC) was calculated by dividing 24hP (g/day) by creatinine content (mmol/day) reported in the results of the same 24 h urine sample

^a^Correlation: negligible (*r* = 0.00–0.29), low (*r* = 0.3–0.49), moderate (*r* = 0.5–0.7), high (*r* = 0.7–0.9), very high (*r* = 0.9–0.99)

^b^Measure of agreement: ICC (2, *k*) ≥0.85 = good reliability (agreement)

^c^Measure of agreement: CCC <0.9 = poor agreement

### Agreement between PCR and 24hP/24hC

#### Intraclass correlation coefficients (2, k)

For all urine samples, ICC was 0.87; for group 1, ICC was 0.52; for group 2, ICC was 0.57; for group 3, ICC was 0.73; and for group 4, ICC was 0.77. The agreement was appropriate for all the urine samples; however, it was poor (<0.85) for groups 1, 2, 3, and 4 (Table 2), indicating less than appropriate agreement of paired urine samples in the same group and poor reproducibility of the measures for each group.

#### Concordance correlation coefficients

For all urine samples, CCC was 0.85; for groups 1, 2, 3 and 4, CCCs were 0.62, 0.34, 0.55, and 0.44, respectively. The agreement was poor (<0.9) for all urine samples and for groups 1, 2, 3, and 4 (Table 2), indicating that the PCR levels were not equivalent to the corresponding 24hP levels in the same patient. The CCC results stratified by ethnicity were as follows: white 0.69 (95 % CI 0.65–0.74), black 0.94 (95 % CI 0.92–0.96), Asian 0.73 (95 % CI 0.63–0.82), and other ethnicities 0.69 (95 % CI 0.60–0.77).

#### Bland-Altman plot

**Fig. 2:**
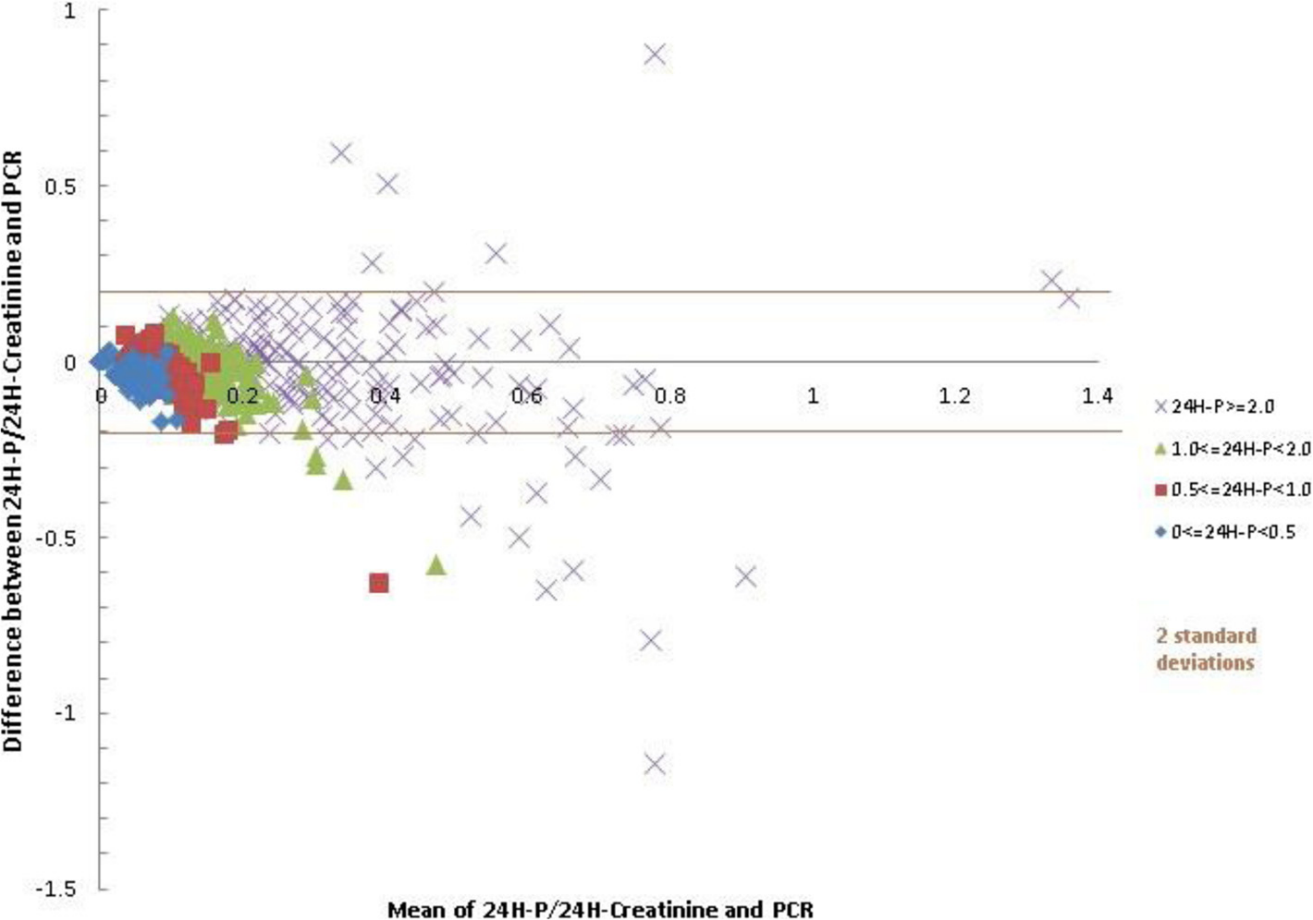
Bland-Altman plot for ratios of protein content and creatinine content in 24-h urine collection samples (24H-P/24-H Creatinine) and untimed samples of urine protein/creatinine ratio (PCR)

The Bland-Altman plot showed that all the paired urine samples from group 1 were between the limits of agreement; however, with increases in 24hP values, a large difference between the two methods was found that was more obvious in groups 3 and 4 than in the other groups. In group 4, PCR tended to overestimate the 24hP results. On the Bland-Altman plot, the dots corresponding to groups 3 and 4 are outside 2 SD, signifying poor agreement (Fig. 2).

### Cutoff of PCR predicting a 24hP of 0.5, 1 and 2 g/day

**Fig. 3:**
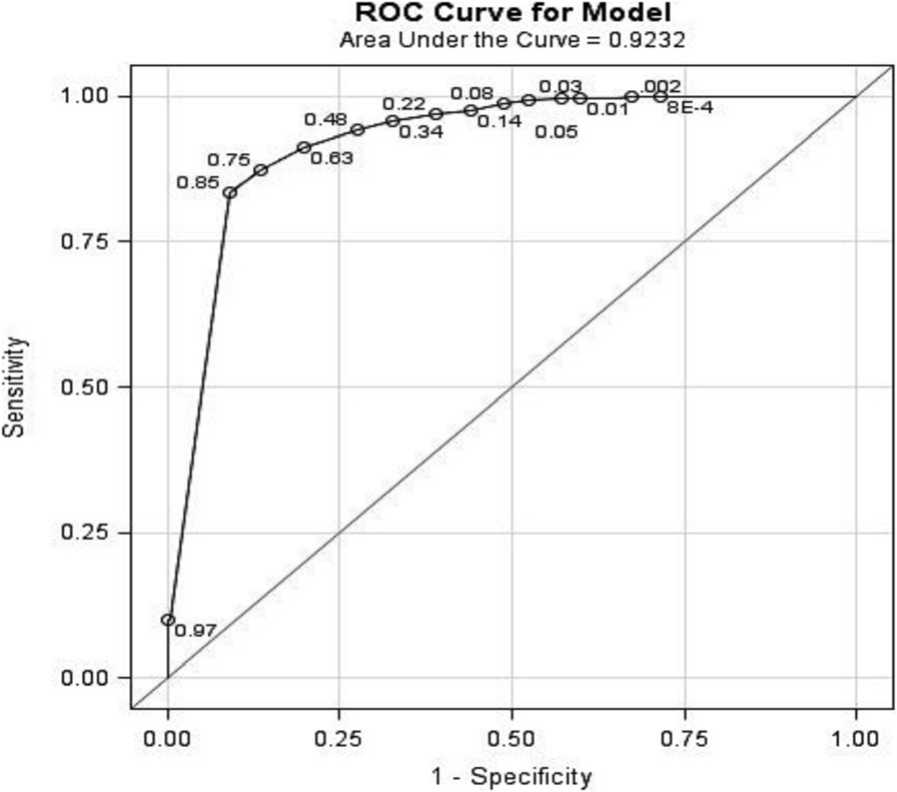
Receiver operating characteristic (ROC) curve for best cutoff of untimed samples of urine protein/creatinine ratio using binary protein content in a 24-h urine collection sample (24hP) as the gold standard (24hP cutoff 0.5 g/day)

The contingency table, using 24hP of 0.5 g/day as the gold standard and PCR of 0.05 g/mmol as the index test, showed Sn and Sp values of 91 % and 83 %, respectively. The results of the ROC curve derived from the logistic regression showed that a PCR of 0.08 g/mmol (800 mg/g) reflected 24hP of 0.5 g/day, with Sn of 91 % and Sp of 80 % (Table 3). The overall area under the curve was 0.92 (Fig. 3). For the 24hP cutoffs of 1.0 and 2.0 g/day, the analyses showed PCR values of 0.16 g/mmol (1600 mg/g; Sn 90 % and Sp 83 %) and 0.35 g/mmol (3500 mg/g; Sn 91 % and Sp 85 %), respectively.

**Table 3:** ROC classification for best PCR cutoffs reflecting 24hP 0.5 g/day using binary 24hP as the gold standard

Probability level	Number of correctly predicted events	Number of correctly predicted nonevents	Number of nonevents predicted as events	Number of events predicted as nonevents	Sensitivity	Specificity	PCR
0.983	65	570	1	597	0.098	**0.998**	0.138
0.970	66	570	1	596	0.100	**0.998**	0.128
0.909	66	569	2	596	0.100	**0.996**	0.108
0.847	552	519	52	110	0.834	**0.909**	0.098
0.754	579	493	78	83	0.875	**0.863**	0.088
**0.628**	**604**	**458**	**113**	**58**	**0.912**	**0.802**	**0.078**
0.340	634	385	186	28	0.958	**0.674**	0.058

*24hP* protein content in a 24-h urine collection sample, *PCR* untimed sample of urine protein/creatinine ratio, *ROC* receiver operating characteristic

Boldface type indicates statistically significant values

### Sensitivity analyses for ACE inhibitors and/or ARB

The results for patients taking ACE inhibitors and/or ARB showed that, correlation among the 164 patients taking ACE inhibitors and/or ARB, 624 paired samples were identified. Although the correlation between 24hP and PCR for all samples was 0.77, in group 1 (n = 288) it was 0.4; in group 2 (n = 129), it was 0.2; in group 3 (n = 114), it was 0.3; and in group 4 (n = 95), it was 0.6.

For all urine samples, ICC was 0.91; for groups 1, 2, 3, and 4, ICCs were 0.48, 0.60, 0.64, and 0.84, respectively. For all samples, CCC was 0.93; for groups 1, 2, 3, and 4, CCCs were 0.59, 0.25, 0.56, and 0.85, respectively.

For the 24hP cutoffs of 0.5, 1.0, and 2.0 g/day, the analyses showed PCR values of 0.07 g/mmol (700 mg/g; Sn 91 % and Sp 82 %), 0.11 g/mmol (1100 mg/g; Sn 90 % and Sp 73 %), and 0.15 g/mmol (1500 mg/g; Sn 90 % and Sp 79 %), respectively.

The analysis for patients not taking ACE inhibitors and/or ARBs showed results similar to those for the patients taking ACE inhibitors and/or ARBs. CCC was 0.54 for all urine samples overall; for groups 1, 2, 3, and 4, CCCs were 0.54, 0.36, 0.39, and 0.36, respectively.

## Discussion

In daily practice, proteinuria in patients with SLE has great importance for the diagnosis of LN, monitoring of disease activity, and prognosis [6, 8]. Thus, it is very important to use the most accurate method for measurement of proteinuria. 24hP is the gold standard for proteinuria assessment in LN, but PCR has become a widely accepted method of measurement of proteinuria in clinical practice, research settings, and clinical trials [9, 36].

The expected creatinine content for different ethnic groups was similar in our patients, without abnormal results for any group. Besides this, whereas the correlation overall of all the paired urine samples and for the ethnic groups was high, it decreased for the individual groups, especially those with higher levels of proteinuria. It is remarkable that the correlation was negligible for the 24hP 0.5–0.99 g/day group, in which this amount of protein excretion should alert the clinician about kidney involvement in the course of SLE. For the groups with higher levels of proteinuria (24hP 1–1.99 and ≥2.0 g/day), although the correlations improved, they did not reach a high threshold, making it difficult to confirm that PCR can replace 24hP for diagnostic purposes. Leung et al. also showed a high correlation for all samples, but correlation dropped with larger levels of proteinuria [20].

Correlation and agreement are two different concepts for a new test. To replace a gold standard test, the new test has to demonstrate agreement with the gold standard. In our study, we used different statistical methods to study agreement. Although the ICC for all the paired urine samples was appropriate, it was poor for the four independent groups. The CCC showed poor agreement for all the samples and in each of the four groups. The Bland-Altman plot confirmed the lack of agreement between PCR and 24hP, in particular for proteinuria >1 g/day. These results support the findings of poor diagnostic performance of PCR for the studied 24hP groups and emphasize the limited utility of PCR as a substitute for 24hP.

Salesi et al. found a high correlation between 24hP and PCR in 74 female patients [37]. Chitalia et al. pointed out that correlation analysis does not enable a reliable decision to be made in order to replace one with the other [38]. Bland and Altman noted that the correlation is described over the entire sample range and may conceal disagreement between two tests at the extreme ranges of the same sample, and it is possible to have a high correlation with prediction intervals that may be unacceptably wide [39]. Birmingham et al. studied 64 patients with SLE and showed a moderate correlation and weak agreement for samples between 0.5 and 3.0 g/day [18]. Choi et al. studied 102 patients and found poor agreement for the group with 24hP 0.5–3 g/day [13]. Zhang et al. found poor agreement in a study of 90 samples [23]. Conclusions based on correlation alone supported the substitution of 24hP by PCR, whereas studies with more appropriate statistical analyses involving agreement clearly showed a weak agreement between both tests. Our study, supported by the use of appropriate statistical analyses, emphases the inadequate validity of PCR as a surrogate for 24hP.

We calculated the best cutoff of PCR predicting a 24hP of 0.5, 1, or 2 g/day. To decide on the best combination of Sn and Sp for a screening test, it is important to consider the costs and possible harms associated with the test [40]. The ROC curve showed that PCR of 0.08, 0.16, or 0.35 g/mmol would reflect 24hP of 0.5, 1, or 2 g/day, respectively. Leung et al. [20] determined the PCR cutoffs for 24hP of 0.5, 1, and 3.5 g/day in 129 samples from 82 patients, and their results (0.45, 0.7, and 1.85 mg/mg, respectively) were different from ours. Some of the patients in Leung et al.’s study did not complete the 24-h urine collection, causing the Sn and Sp to be underestimated. In our study, all the paired samples were available, and our sample size was larger. PCR is a test used for clinical decision-making, but when the quantification of protein is needed, the 24hP still is the gold standard.

In the present study, the Sn analyses conducted on patients maintained on ACE inhibitors and/or ARBs, as well as on patients not taking ACE inhibitors and/or ARBs, also showed poor reproducibility of the results for 24hP by PCR; thus, PCR cannot be substituted for 24hP.

Our study has some limitations. The proteinuria measurement tests (24hP and PCR) were not performed on the same day. However, in this analysis, both tests were performed in less than a 4-day period, which is considered too short to affect the confidence of the proteinuria measurement. Group 4 had a relatively small sample size (43 patients with 174 paired urine samples), thus limiting the generalizability of our findings. However, our sample size was bigger than the samples in other studies with similar findings [18]. We conducted a cross-sectional study, and thus analyses of serial samples are not presented. This is a very relevant question that needs to be answered in future studies and may be more suitable in an incident cohort of patients with lupus.

## Conclusions

Clinicians in daily practice managing SLE and LN rely on the accuracy and validity of laboratory results to make appropriate decisions, such as ordering a kidney biopsy or initiating and/or modifying treatment for LN based on proteinuria level. On the basis of the results of our study, we conclude that PCR can be used as screening test but still lacks the performance level of the gold standard test, 24hP, in measuring proteinuria. The importance of this finding is that clinicians cannot rely on PCR results to make treatment decisions. PCR can be used as a screening test in patients with SLE, but every abnormal result should be confirmed with a 24-h urine collection sample to detect the precise level of proteinuria.

## Abbreviations

ACE: Angiotensin-converting enzyme
ACR: American College of Rheumatology
ARB: Angiotensin receptor blocker
CCC: Concordance correlation coefficient
CI: Confidence interval
ExC: Expected creatinine content in a 24-h urine sample collection
GFR: Glomerular filtration rate
24hC: Measured creatinine content in a 24-h urine sample collection
24hP: Protein content in a 24-h urine collection sample
24hP/24hC: Ratio of the protein content and the creatinine content in a 24-h urine collection sample
ICC: Intraclass correlation coefficient
LN: Lupus nephritis
LR+: Positive likelihood ratio PCR, Untimed sample of urine protein/creatinine ratio
ROC: Receiver operating characteristic
SD: Standard deviation
SLE: Systemic lupus erythematosus
SLEDAI-2K: Systemic Lupus Erythematosus Disease Activity Index 2000
SD: Standard deviation
Sn: Sensitivity
Sp: Specificity
PCR: Spot urine protein/creatinine ratio
